# Efficacy and safety of eradication therapy for elderly patients with helicobacter pylori infection

**DOI:** 10.1097/MD.0000000000016619

**Published:** 2019-07-26

**Authors:** Satoshi Kobayashi, Satoru Joshita, Chikara Yamamoto, Takumi Yanagisawa, Takayuki Miyazawa, Megumi Miyazawa, Daisuke Kubota, Junichi Sato, Takeji Umemura, Eiji Tanaka

**Affiliations:** a Department of Gastroenterology, Hokushin General Hospital, Nagano Prefectural Federation of Agricultural Cooperatives for Health and Welfare, Nakano; b Division of Gastroenterology and Hepatology, Department of Medicine, Shinshu University School of Medicine; c Research Center for Next Generation Medicine, Shinshu University, Matsumoto, Japan.

**Keywords:** adverse events, elderly patients, eradication, *Helicobacter pylori (H. pylori)*, safety

## Abstract

*Helicobacter pylori* (*H. pylori*) is the most prevalent chronic bacterial infection and is associated with chronic gastritis, peptic ulcer disease, and gastric adenocarcinoma. Although eradication therapy is widely performed for *H. pylori* infection, adverse events (AEs) are of particular concern in the elderly. This study investigated the efficacy and safety of *H. pylori* eradication therapy for elderly patients.

Retrospective investigation of 1271 cases (median age: 61 years, 730 male) of *H. pylori* infection was performed to compare clinical indications and outcomes among the younger group (<65 years old), elderly group (65–74 years old), and super-elderly group (>75 years old).

Chronic gastritis (77.0%) and gastric and/or duodenal ulcer (16.4%) were the most frequent indications for eradication therapy in the cohort. The respective eradication and AE rates for the first and second treatment regimens were 92.1% (1044 of 1133 cases) and 9.1% (103 of 1133 cases) and 84.2% (123 of 146 cases) and 8.9% (13 of 146 cases). No significant differences were detected for eradication rate or AE frequency between the super-elderly group and the other groups. Prior to therapy, the super-elderly group had significantly less frequent chronic gastritis than the other groups but more frequent gastric or duodenal ulcer and post-gastric cancer treatment (all *P* < .001), indicating a reluctance for clinicians to treat very old patients, possibly due to unfounded concerns of complications.

Triple therapy for *H. pylori* eradication is effective and safe, even for elderly patients.

## 1 Introduction

*Helicobacter pylori* (*H. pylori*) was firstly reported by Warren and Marshall in 1983 as a gram-negative bacterium found on the luminal surface of the gastric epithelium.^[1]^ Conservative estimates suggest that half of the world's population is infected with *H. pylori*,^[2]^ although country-specific prevalences appear to decline with economic improvement. For example, 70 to 80% of Japanese adults born before 1950 were infected by the bacterium, versus 45% for those born between 1950 and 1960 and 25% for those born between 1960 and 1970.^[3]^ This rapid fall has been attributed to Japan's post-war economic progress and ameliorations in sanitation. However, many elderly patients remain infected with *H. pylori* until receiving eradication therapy.

*H. pylori* is known to cause chronic gastritis, gastric and duodenal ulcers, gastric adenocarcinoma, and gastric mucosa-associated lymphoid tissue (MALT) lymphomas.^[4–6]^ In addition to symptomatic treatment, *H. pylori* eradication also appears to reduce the risk of gastric cancer,^[7]^ especially metachronous gastric cancer,^[8]^ and enhance gastric corpus atrophy grade improvement from baseline.^[9]^ Therefore, all individuals with evidence of active *H. pylori* infection should be offered with eradication therapy in the absence of screening programs for asymptomatic cases.

Several antibiotic regimens for *H. pylori* eradication therapy have been confirmed as effective and safe. ^[10]^The adverse events (AEs) of eradication therapy are usually mild, with fewer than 10% of patients halting treatment due to unwanted reactions.^[11]^ However, AE rates have been as high as 50% for a particular triple therapy regimen.^[11,12]^ The frequency of AEs induced by *H. pylori* eradication therapy in the elderly also remains unclear and possibly misunderstood. This study therefore investigated the efficacy and safety of *H. pylori* eradication therapy for elderly patients.

## 2 Patients and methods

### 2.1 Patients

A total of 1276 patients who had received *H. pylori* eradication therapy between January 2013 and December 2017 were initially recruited in this retrospective, single-center study. After excluding cases lacking sufficient clinical data for analysis, 1271 patients were ultimately enrolled. The cohort was divided by age into the younger group (≤65 years), elderly group (65–74 years), and super-elderly group (≥75 years) based on the standards of the Japanese national health insurance system for comparisons of clinical characteristics including indication for eradication, eradication regimen, eradication rate, and rate and type of AEs.

### 2.2 Confirmation of *H. pylori* positivity and laboratory testing

*H. pylori* infection was confirmed by any positive result among serum anti-*H. pylori* antibodies, urea breath test, stool antigen test, and histopathology. All patients also underwent esophagogastroduodenoscopy (EGD) before eradication therapy to confirm the presence of active chronic gastritis.

Serum anti-*H. pylori* IgG antibody titer was measured using an enzyme immunoassay, with a level of >10 U/mL defined as positive (SRL, Inc., Tokyo, Japan). The urea breath test was performed using infrared spectroscopic analysis according to the manufacturer's instructions (BML, Inc., Tokyo, Japan). The stool antigen test was carried out using an enzyme-linked immunosorbent assay (SRL, Inc., Tokyo, Japan). Cases were histopathologically defined as *H. pylori* positive when bacterial bodies were evident in EGD-obtained tissue sections.

### 2.3 Treatment regimens for eradication

Patients were initially treated with a regimen of clarithromycin (CAM)-based triple therapy consisting of a proton pump inhibitor (PPI), amoxicillin (AMPC), and CAM for 7 days as a first line treatment. Unresponsive patients identified 4 to 8 weeks later by means of the urea breath test were immediately given PPI, AMPC, and metronidazole (MNZ) as a second regimen. Two-week regimens are currently not approved in Japan. Successful eradication was confirmed by a urea breath test performed 4 weeks or more after completion of triple antibiotic therapy.

### 2.4 Determination of AEs

AEs including diarrhea, eruptions, and constipation (Table 2) were retrospectively determined by chart reviews of medical records.

### 2.5 Statistical analysis

Statistical analysis was carried out using StatFlex ver. 7.0.2 (Artech Co., Ltd., Osaka, Japan). Data were presented as the median ± interquartile range for continuous variables, which were compared using the Mann–Whitney *U* test. Categorical variables were presented as the frequency (percentage) and analyzed using the chi-square test. All statistical tests were two-sided and evaluated at the 0.05 level of significance.

### 2.6 Ethics of the study

This investigation was reviewed and approved by the Institutional Review Board of Hokushin General Hospital (Nakano, Japan) (approval number: 2018021). As this was a retrospective observational study, written informed consent was not obtained from participating subjects, although study information was presented in an opt-out format at the hospital. This investigation was conducted in accordance with the principles of the 1975 Declaration of Helsinki as revised in 1983.

## 3 Results

### 3.1 Clinical characteristics of enrolled patients

**Figure 1:**
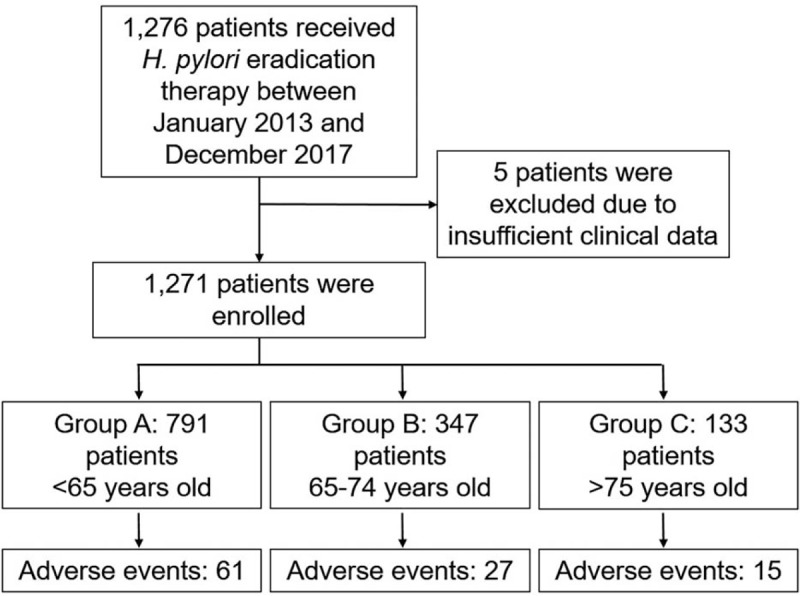
Study flowchart.

A flowchart of this study is shown in Fig. 1. The clinical characteristics of the enrolled patients are summarized in Table 1. Median age was 61 years and 57.4% of the cohort was male. Chronic gastritis (77.0%) was the most frequent indication for eradication therapy, followed next by gastric and/or duodenal ulcer with chronic gastritis (16.4%) and post-gastric cancer treatment with chronic gastritis (5.4%). The vast majority of patients were treated with lansoprazole + AMPC + CAM or vonoprazan + AMPC + CAM for the first line treatment (Table 1). The eradication rate of the first regimen was 92.1% (1044 of 1133 cases), with AEs seen in 9.1% (103 of 1133) of cases. The eradication rate of the second regimen was 84.2% (123 of 146 cases), with AEs recorded in 8.9% (13 of 146) of cases. The most frequently encountered AEs were diarrhea (51.7%), eruptions (12.9%), and constipation (7.8%). Fourteen patients halted treatment due to eruptions during the first regimen (Table 2).

**Table 1:**
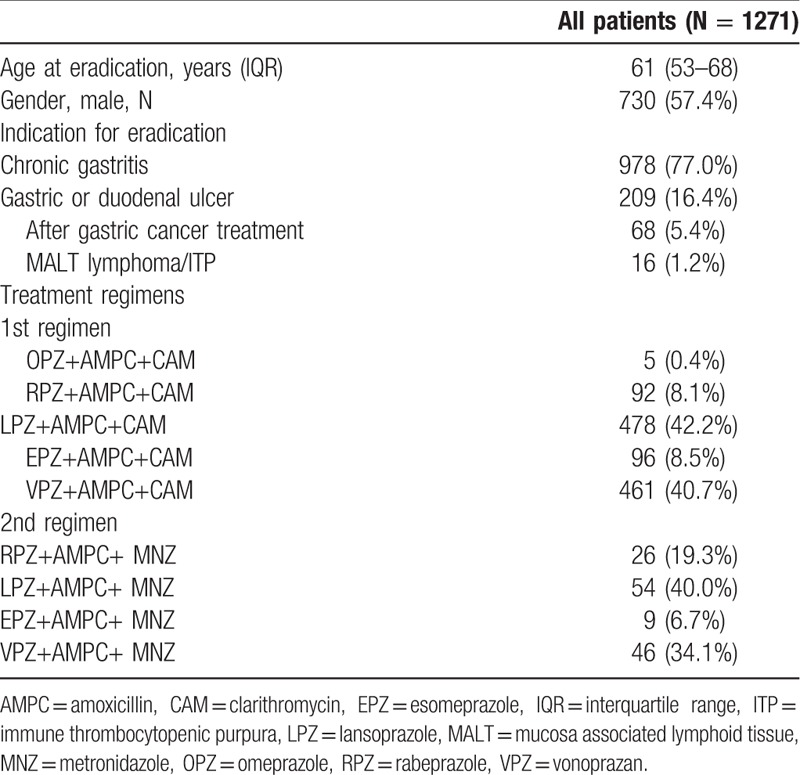
Clinical characteristics of enrolled patients.

**Table 2:**
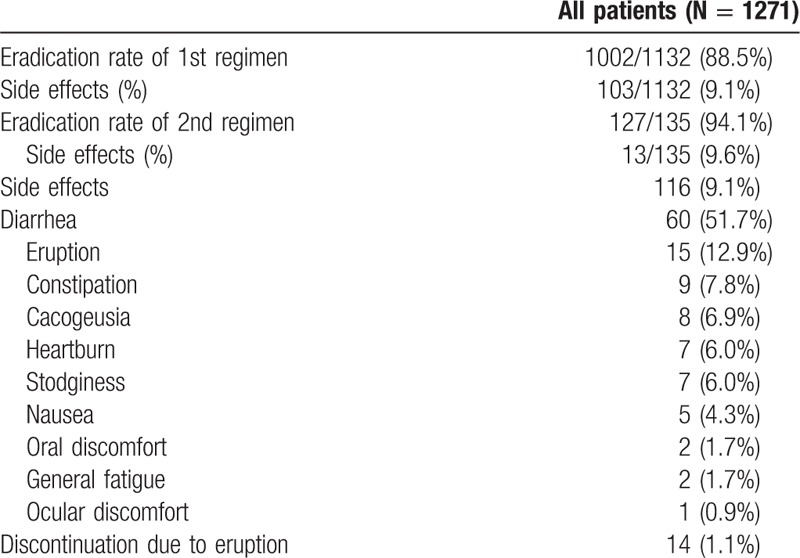
Treatment outcomes and adverse events.

### 3.2 Comparisons among age groups

Clinical characteristics were compared among the younger, elderly, and super-elderly groups (Table 3). Super-elderly patients had a significantly less frequent indication of chronic gastritis than did the other groups (both *P* < .001) but more frequent indications of gastric or duodenal ulcer (both *P* < .001) and post-gastric cancer treatment (both *P* < .001). Apart from a significant difference between the younger and elderly groups for first eradication rate (*P* < .05), no remarkable differences were seen among the groups for the efficacy of either regimen. Moreover, no significant differences were observed in comparisons of AE rates among the groups (Table 4). The incidence of AEs was comparable between the first and second regimens.

**Table 3:**
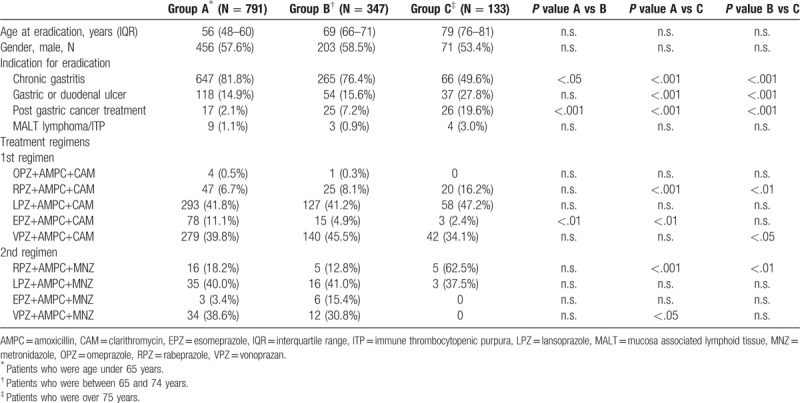
Clinical characteristics of the three groups.

**Table 4:**
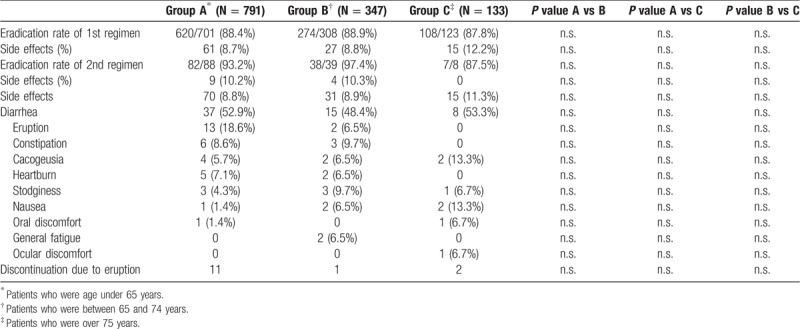
Treatment results of the three groups.

## 4 Discussion

This study uncovered two clinically significant points:

1. the eradication rates of *H. pylori* for very elderly patients were not inferior to those of younger patients, and

2. the AE rates of eradication therapy were comparable among all age groups.

Our findings confirm that eradication therapy is effective and safe for *H. pylori*, even for patients at an advanced age.

We divided the cohort of this retrospective study into three groups (<65 years old, 65–75 years old, and >75 years old) based on the age tiers established by the Japanese national health insurance system. Patients with active *H. pylori* infection were indicated for treatment, with the vast majority receiving eradication therapy due to chronic gastritis associated with chronic infection. Interestingly, less than half of the super-elderly patients had undergone eradication therapy, which indicated a possible reluctance among clinicians to prescribe such treatment. One reason might have been out of consideration for patient age and a higher perceived risk of complications. However, this study revealed no differences between the super-elderly and other groups in terms of eradication rates and AEs, suggesting that clinicians need not withhold treatment strictly based on age.

The infection route of *H. pylori* remains unknown, although person-to-person transmission through fecal/oral or oral/oral exposure seems most likely.^[13,14]^ Person-to-person transmission has been supported by intrafamilial clustering of *H. pylori* infection, in which infected individuals were more likely to have infected spouses and children than were uninfected individuals.^[15,16]^ Accordingly, our data highlight the importance of elderly or super-elderly patients who are infected with *H. pylori* to receive eradication therapy for prevention of cross-infection to younger uninfected family members, especially in multigenerational households.

As expected, the frequency of AEs in our cohort was <10%. The most frequent culprits, diarrhea and constipation, are manageable with drugs and probiotics. It was reported that supplementation with probiotics was effective not only in decreasing eradication therapy-related AEs, but also in increasing eradication rate.^[17]^ Thus, *H. pylori* eradication triple therapy along with probiotics might improve both patient treatment and comfort.

This study has several limitations. First, it was conducted as a single-center cohort study of approximately 1000 patients. Secondly, due to its retrospective design, we cannot exclude the possibility that only elderly patients with a favorable systemic condition received eradication therapy.

## 5 Conclusions

Triple therapy for *H. pylori* eradication was effective and safe for elderly patients. Further studies are needed to examine the longer-term clinical effects of eradication therapy, such as the prevention of peptic ulcer recurrence, in larger, multi-center, elderly cohorts.

## Acknowledgments

The authors would like to thank Trevor Ralph for his English editorial assistance.

## Author contributions

**Conceptualization:** Satoshi Kobayashi.

**Data curation:** Satoshi Kobayashi, Satoru Joshita.

**Formal analysis:** Satoshi Kobayashi.

**Methodology:** Satoshi Kobayashi, Chikara Yamamoto.

**Project administration:** Satoshi Kobayashi, Satoru Joshita.

**Resources:** Satoshi Kobayashi, Chikara Yamamoto, Takumi Yanagisawa, Takayuki Miyazawa, Megumi Miyazawa, Daisuke Kubota, Junichi Sato.

**Software:** Satoshi Kobayashi.

**Supervision:** Eiji Tanaka.

**Validation:** Satoshi Kobayashi, Satoru Joshita, Takeji Umemura.

**Visualization:** Satoshi Kobayashi, Satoru Joshita.

**Writing – original draft:** Satoshi Kobayashi, Satoru Joshita.

**Writing – review & editing:** Chikara Yamamoto, Takeji Umemura, Eiji Tanaka.

Satoru Joshita orcid: 0000-0002-6364-9654.
